# Supplementary material for: Game Birds Can Act as Intermediaries of Virulent Genotype VII Avian Orthoavulavirus-1 between Wild Birds and Domestic Poultry
Source: Viruses. 2023 Feb 14;15(2):536. doi: 10.3390/v15020536 (PMC9968179; doi:10.3390/v15020536)
Supplement: Supplementary file 1 [file viruses-15-00536-s001.zip › viruses-2210149-supplementary.pdf]

Supplementary Figure

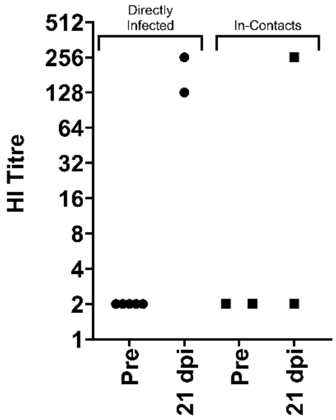

Figure S1. Sero-conversion of directly infected and in-contact partridges with APMV-1/chicken/Bulgaria/112/13. Sera was recovered pre-infection and at the termination of the experiment of available partridges. HI titres were determined by use of homologous antigen and are expressed as log_2 reciprocal titre.
